# Recent advances in the treatment of pain in endometriosis: A bibliometric analysis of experimental models

**DOI:** 10.14202/vetworld.2023.2329-2339

**Published:** 2023-11-27

**Authors:** Rafael Abreu Lima, Isabela Bastos Jácome Souza, Gustavo Medeiros Frota, Tereza Cristina Monteiro Melo Prazeres, Ingrid Campos Albuquerque, Eduardo Martins de Sousa, Maria do Socorro Sousa Cartagenes, Rafael Cardoso Carvalho, Adalgisa Sousa Paiva Ferreira, João Batista Santos Garcia

**Affiliations:** 1 Graduate Program in Health Sciences, Center for Biological and Health Sciences, Federal University of Maranhão, São Luís, 65085-580, Brazil; 2 Northeast Biotechnology Network, Center for Biological and Health Sciences, Federal University of Maranhão, São Luís, 65085-580, Brazil; 3 Department of Nursing, Center for Biological and Health Sciences, Federal University of Maranhão, São Luís, 65085-580, Brazil; 4 Graduate Program in Microbial Biology, CEUMA University, São Luís, 65-75-120, Brazil

**Keywords:** animal model, animal science, endometriosis, rodents, treatments

## Abstract

**Background and Aim:**

Treatment of endometriosis involves pain relief which is achieved through the administration of analgesics and non-steroidal anti-inflammatory drugs, with or without the addition of hormone therapy. At present, studies investigating endometriosis pain management using experimental rat models and the use of medications are scarce. Therefore, this study aimed to systematically evaluate research trends and critical points in the field of endometriosis pain management using experimental models.

**Materials and Methods:**

A total of 30 publications related to this topic that were published from 2012 to 2022 were retrieved from various databases, including Web of Science, Scopus, PubMed, Embase, and CINAHL, using appropriate English keywords. The quality of the publications was evaluated using impact metrics, productivity, term density mapping, and author network.

**Results:**

The average publication rate was three articles per year, reaching its peak in 2021 at five articles per year. The United States and China were found to be the most productive countries, with 12 and 10 publications per year, respectively. The field of medicine (37.0%) was the most abundant, although the H-index was relatively low (13:13). Term density mapping involved the search of 542 keywords, of which 35 were selected, with only 8 exhibiting significant density.

**Conclusion:**

In the past decade, there has been a shortage of publications that have addressed pain in endometriosis in experimental models and, within this context the majority of the production and publication in this field has been performed by the United States and China. After performing this bibliometric review, it can be inferred that more research is required in this field, to develop new approaches and treatments for endometriotic pain.

## Introduction

Endometriosis is a chronic, inflammatory, benign, and estrogen-dependent gynecological disease. It is characterized by the aberrant growth of endometrial tissue outside the uterine cavity. Endometriosis affects approximately 10% of women in their reproductive years and is associated with 50% of infertility cases [1, 2]. Due to its multifactorial etiology, chronic pelvic pain occurs in more than 70% of cases, which is often accompanied by debilitating dyspareunia, dysmenorrhea, and other symptoms, negatively affecting the patient’s daily activities and overall quality of life. The diagnosis of endometriosis is time-consuming, and conventional drug treatments are expensive and often exhibit limited efficacy, with potential for relapse and complications on treatment cessation [3, 4].

Therefore, regardless of the therapeutic approach used, it is necessary to develop a treatment strategy that aligns with each patient’s characteristics and ensures easy therapeutic adherence [5]. In this regard, animal models have been extensively used in experimental studies on endometriosis to enhance our understanding of its pathogenesis, progression, and potential treatment modalities [6]. However, existing knowledge in this field is still insufficient to provide conclusive guidance, particularly regarding the currently proposed treatments [7]. Furthermore, several gaps still need to be elucidated, particularly in studies investigating endometriosis pain management using experimental rat models and exploring the use of non-conventional medications.

To address these knowledge gaps, a bibliometric analysis was proposed as it represents a well-established method for evaluating research trends using quantitative and qualitative measurements. Using bibliometric indicators, such as the temporal distribution of studies, number of authors, publications, countries, scientific collaborations, journals, and impact factors, this analysis aimed to provide comprehensive insights into the progress of scientific research and technological advancements in the field [8, 9].

Despite the growing number of annual publications, a comprehensive bibliometric analysis focusing on this area has not yet been conducted. Thus, this study was conducted to systematically evaluate research trends and critical points in the field of endometriosis pain management using experimental rat models, proposing a new paradigm for future research.

## Materials and Methods

### Ethical approval

Since this study involved the analysis of existing published research, it did not require ethical approval. The data used were publicly available and did not involve any direct interaction with human subjects or their personal information. In addition, no authors were contacted for additional information about their publications, as the analysis focused solely on the existing data within the publications themselves.

### Study period and location

Data were collected independently by pairs from January to February 2023. The data were extracted at Federal University of Maranhão, São Luís, Brazil.

### Data sources

The specific topic of interest was endometriosis pain management in experimental rat models, focusing on articles published in indexed journals from January 2012 to December 2022. Multiple databases, namely, Scopus, Web of Science (WOS), CINAHL, Embase, World Health Organization, and PubMed, were searched to obtain relevant data.

Web of Science and Scopus, which are owned by Clarivate and Elsevier, respectively, are widely recognized sources of citation data that are curated by subscribing institutions. The combination of these two databases encompasses a broader range of scientific disciplines and a wider span of publication dates, includes a larger number of countries, and allows for comprehensive citation analysis. Due to these advantages, these databases are commonly used in bibliometric analyses [9, 10].

### Search strategy

A comprehensive search strategy was used to ensure the inclusion of all relevant publications. The keywords used in the search were ([endometriosis] OR [endometriose]) AND ([animal model] OR [animal models] OR [animal model experimental] OR [animal models experimental] OR [animal model laboratory]) AND ([rats] OR [rattus] OR [laboratory rats]) AND ([treatments] OR [management]) AND ([pain] OR [management pain]). Furthermore, the search was restricted to experimental studies published in English.

### Inclusion and exclusion criteria

To refine the search results, the inclusion criterion used was as follows: experimental research articles published from 2012 onward in indexed databases in the English language. The publications were evaluated using various bibliometric indicators: total citation frequency, average citations per item, H-index, CiteScore, SCImago Journal Rank (SJR), and source-normalized impact per paper (SNIP). Furthermore, several factors were considered to analyze the quantity and publication trends, including the total number of publications, types of research conducted, research organizations involved, author contributions, publication journals, and financial support received. Finally, specific information such as the number of publications, citations received, H-index values, publication journals, references, and keywords were obtained and recorded as bibliometric indicators. On the other hand, the exclusion criteria were duplicate publications, dissertations, letters, book chapters, conference proceedings, and articles unrelated to the topic or lacking information on endometriosis pain outcomes. Any disagreements regarding inconsistent contents were discussed and resolved. Articles that could not be adequately assessed for inclusion based on their title alone were also excluded [11]. The citation information for all the included articles was extracted from the Scopus database, ensuring a comprehensive collection of relevant data.

### Bibliometric analysis

This study employed quantitative descriptive analysis based on bibliometric analysis. Data generated from the analysis are presented using graphs and tables, showing the results in absolute numbers and percentages.

VOSviewer® version 1.6.15 (Leiden University, Netherlands) was used to conduct bibliometric mapping and cluster analysis. It is a specialized software tool designed to construct and visualize bibliometric networks. Using bibliometric mapping techniques, the software allows visualization of academic production in terms of publications and citation information within a specific field. In this study, cluster analysis algorithms were used to identify natural divisions, or clusters, within research networks based on similarities. This facilitated the visualization of coauthorship networks among researchers, institutions, and countries [12].

## Results

### Pain management in endometriosis: Publications, authors, affiliations, countries, and grants

**Figure-1:**
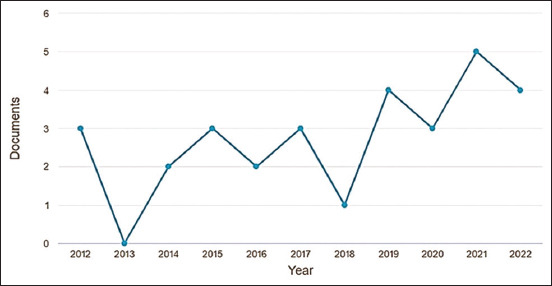
Annual publication trends in pain treatment in endometriosis in experimental rat models, 2012–2022, Bibliometric Review, 2023.

A total of 142 publications (59 from Scopus and 83 from the WOS) were initially identified through database searches. After applying the refinement criteria, including a temporal filter (spanning from 2012 to 2022), document type (articles), and language (English), a final set of 40 reviews (30 from Scopus and 10 from the WOS) was obtained. After excluding 10 duplicate reviews, 30 were finally included in the bibliometric data analysis [13–42] (Figure-1).

The 30 publications included in this analysis are presented in Table-1 [13–42].The United States had the highest number of publications (12), followed by China (10), Turkey (3), and the United Kingdom (2). There were also contributions from Italy, Brazil, Puerto Rico, India, Russia, and Iran, with each having one publication.

**Table-1:** Experimental studies on pain management in endometriosis: publications in experimental rat models, 2012–2022.

Year	Author	Title	Journal	Affiliation of the 1^st^ Author
2012 [13]	Dmitrieva, N., Faircloth, E.K., Pyatok, S., Sacher, F., Patchev, V.	Telemetric assessment of referred vaginal hyperalgesia and the effect of Indomethacin in a rat model of endometriosis	Frontiers in Pharmacology	Program in Neuroscience, Florida State University, Tallahassee, FL, USA
2012 [14]	Zheng, Y., Liu, X., Guo, S.-W	Therapeutic potential of andrographolide for treating endometriosis	Human Reproduction	Shanghai OB/GYN Hospital, Fudan University, Shanghai, China
2012 [15]	Pelch, K.E., Sharpe-Timms, K.L., Nagel, S.C.	Mouse model of induced endometriosis auto-transplantation of uterine tissue	Journal of Visualized Experiments	Obstetrics, Gynecology and Women’s Health and Division of Biological Sciences, University of Missouri, USA
2014 [16]	Alvarez, P., Levine, J.D.	Screening the role of pronociceptive molecules in a rodent model of endometriosis pain	Journal of Pain	Division of Neuroscience, University of California San Francisco, San Francisco, California.USA
2014 [17]	Alvarez, P., Bogen, O., Chen, X., Giudice, L.C., Levine, J.D	Ectopic endometrium-derived leptin produces estrogen-dependent chronic pain in a rat model of endometriosis	Neuroscience	Division of Neuroscience, University of California San Francisco, California, USA.
2015 [18]	Alvarez, P., Giudice, L.C., Levine, J.D.	Impact of surgical excision of lesions on pain in a rat model of endometriosis	European Journal of Pain (United Kingdom)	Division of Neuroscience, University of California San Francisco, California, USA.
2015 [19]	Ray, K., Fahrmann, J., Mitchell, B., (…), Golovko, S., Santanam, N.	Oxidation-sensitive nociception involved in associated pain	Pain	Depts of Pharmacology, Physiology, and Toxicology Biochemistry and Microbiology, and Obstetrics and Gynecology, Joan C. Edwards School of Medicine, Marshall University, Huntington, WV, USA
2015 [20]	Greaves, E., Temp, J., Esnal-Zufiurre, A., (…), Horne, A.W., Saunders, P.T.K.	Estradiol Is a Critical Mediator of Macrophage-Nerve Cross Talk in Peritoneal Endometriosis	American Journal of Pathology	Medical Research Council Center for Reproductive Health, The University of Edinburgh, Queen’s Medical Research Institute, Edinburgh, UK
2016 [21]	Palmer, S.S., Altan, M., Denis, D., (.), Bruner-Tran, K.L., Nataraja, S.G.	Bentamapimod (JNK inhibitor AS602801) induces regression of	Reproductive Sciences	EMD Serono Research Institute, Billerica, MA, USA
2016 [22]	Di Paola, R., Fusco, R., Gugliandolo, E., (…), Granese, R., Cuzzocrea, S.	Co-micronized palmitoylethanolamide/polydatin treatment causes endometriotic lesion regression in a rodent model of surgically-induced endometriosis	Frontiers in Pharmacology	Department of Chemical, Biological, Pharmaceutical and Environmental Sciences, University of Messina, Italy.
2017 [23]	Lian, Y.L., Cheng, M.J., Zhang, X.X., Wang, L.	Elevated expression of transient receptor potential vanilloid type 1 in dorsal root ganglia of rats with endometriosis	Molecular Medicine Reports	Obstetrics and Gynecology Hospital, Fudan University, Shanghai, China
2017 [24]	Dogru, H.Y., Isguder, C.K., Arici, A., (…), Delibas, I.B., Cakmak, B.	Effect of amygdalin on the treatment and recurrence of endometriosis in an experimental rat study	Periodicum Biologorum	Department of Obstetrics and Gynecology, School of Medicine, Gaziosmanpasa University, Turkey
2017 [25]	Yuan, M., Ding, S., Meng, T., (…), Yuan, H., Hu, F.	Effect of A-317491 delivered by glycolipid-like polymer micelles on endometriosis pain	International Journal of Nanomedicine	Institute of Marine Biology, Ocean College, Zhejiang University, Zhoushan, China
2018 [26]	Torres-Reverón, A., Rivera-Lopez, L.L., Flores, I., Appleyard, C.B.	Antagonizing the corticotropin releasing hormone receptor 1 with antalarmin reduces the progression of endometriosis	PLoS ONE	Department Neuroscience, University of Texas at Rio Grande Valley School of Medicine, Edinburgh, Texas, USA
2019 [27]	Gomes Pereira, F.E.X., Medeiros, F.D.C., Rocha, H.A.L., da Silva, K.S.	Effects of omega-6/3 and omega-9/6 nutraceuticals on pain and fertility in peritoneal endometriosis in rats	Acta Cirurgica Brasileira	Department of Surgery, Federal University of Ceará (UFC), Fortaleza-Ce, Brazil
2019 [28]	Zhu, T.H., Zou, G., Ding, S.J., (…), Yao, Y.X., Zhang, X.M.	Mast cell stabilizer ketotifen reduces hyperalgesia in a rodent model of surgically-induced endometriosis	Journal of Pain Research	Department of Obstetrics and Gynecology, Women’s Hospital, Zhejiang University School of Medicine, Hangzhou, China
2019 [29]	Warriar, P., Barve, K., Prabhakar, B.	Anti-arthritic effect of garcinol enriched fraction against adjuvant induced arthritis	Recent Patents on Inflammation and Allergy Drug Discovery	School of Pharmacy and Technology Management, SVKM’s NMIMS, Mumbai, Maharashtra, India.
2019 [30]	Ge, P., Ren, J., Harrington, A.M., (…), Brierley, S.M., Hannig, G.	Linaclotide treatment reduces endometriosis-associated vaginal hyperalgesia and mechanical allodynia through viscero-visceral cross-Talk	Pain	Ironwood Pharmaceuticals, Cambridge, MA, USA
2020 [31]	Yarmolinskaya, M., Khobets, V., Tral, T., Tkachenko, N.	The potentialities of oxytocin receptor inhibitors for endometriosis therapy	Gynecological Endocrinology	Dept of Gynecology and Endocrinology, FSBSI, Saint Petersburg, Russia.
2020 [32]	Tian, F., Cheng, W., Hu, J., Huang, S., Sun, S.	Effects of botulinum toxin A on endometriosis-associated pain and its related mechanism	Molecular Medicine Reports	Dept of Anesthesiology, Obstetrics and Gynecology Hospital of Fudan University, Shanghai, China
2020 [33]	Seguinot-Tarafa, Inevy; Luna, Núria; Suárez, Edu; Appleyard, Caroline B.; Flores, Idhaliz	Inhibition of Histone Methyltransferase EZH2 Suppresses Endometriotic Vesicle Development in a Rat Model of Endometriosis	Reproductive Sciences	Dept of Basic Sciences, Ponce Research Institute, Ponce Health Sciences University, Puerto Rico
2021 [34]	Wang, C., Chen, Z., Zhao, X., (…), Guan, M.X., Xi, Y.	Transcriptome-based analysis reveals therapeutic effects of resveratrol on endometriosis in arat model	Drug Design, Development and Therapy	The Women’s Hospital, Zhejiang University School of Medicine, Hangzhou, Zhejiang, China
2021 [35]	Zhang, W., Wang, J., Wu, J., (…), Liang, X., Cao, L.	Exploration of the Modulatory Property Mechanism of ELeng Capsule in the Treatment of Endometriosis Using Transcriptomics Combined with Systems Network Pharmacology	Frontiers in Pharmacology	The Second Clinical College of Guangzhou University of Chinese Medicine, Guangzhou, China
2021 [36]	Su, W., Cui, H., Wu, D., (…), Huang, Y., Ma, C.	Suppression of TLR4-MyD88 signaling pathway attenuated chronic mechanical pain in a rat model of endometriosis	Journal of Neuroinflammation	Dept of Anesthesiology, Peking Union Medical College Hospital, Chinese Academy of Medical Sciences and Peking Union Medical College, Beijing, China.
2021 [37]	Farahani, Z.K., Taherianfard, M., Naderi, M.M., Ferrero, H.	Possible therapeutic effect of royal jelly on endometriotic lesion size, pain sensitivity, and neurotrophic factors in a rat model of endometriosis	Physiological Reports	Physiology Division of Basic Sciences Department, School of Veterinary Medicine, Shiraz University, Shiraz, Iran.
2021 [38]	Davenport, A.J., Neagoe, I., Bräuer, N., (…), Zollner, T.M., Fischer, O.M.	Eliapixant is a selective P2X3 receptor antagonist for the treatment of disorders associated with hypersensitive nerve fibers	Scientific Reports	Discovery Chemistry, Dorothy Crowfoot Hodgkin Campus, Evotec UK, United Kingdom.
2022 [39]	Kaplan, S., Kırıcı, P., Türk, A.	The effects of adalimumab on the rat auto-transplantation endometriosis model: A placebo-controlled randomized study	Advances in Clinical and Experimental Medicine	Faculty of Medicine, Adıyaman University, Turkey.
2022 [40]	Akkol EK, Karpuz B, Türkcanoğlu G, Coşgunçelebi FG, Taştan H, Aschner M, Khatkar A, Sobarzo-Sánchez E	The Phytochemical Profile and Biological Activity of Malva neglecta Wallr in Surgically Induced Endometriosis Model in Rats	Molecules	Department of Pharmacognosy, Faculty of Pharmacy, Gazi University Turkey
2022 [41]	Qin Z, Dong Z, Liu J, Zhong A, Bao M, Wang H, Yu H, Zhang S, Zhang W, Shen L, Wu J, Chen J.	A Preliminary Study on the Effects of Black Cohosh Preparations on Bone Metabolism of Rat Models with GnRH-a-Induced Peri-Menopausal Symptoms	Front Endocrinol (Lausanne)	Department of Obstetrics and Gynecology, The Affiliated Changzhou No. 2 People’s Hospital of Nanjing Medical University, Changzhou, China
2022 [42]	Zong C, Sun L, Xu X, Xue X.	Huayu Sanjie Enema Liquid Relieves Pain in Endometriosis Model Rats by Inhibiting Inflammation, Peripheral Sensitization, and Pelvic Adhesion	Evid Based Complement Alternate Med	Dongzhimen Hospital, Beijing University of Chinese Medicine, Beijing, China

**Figure-2:**
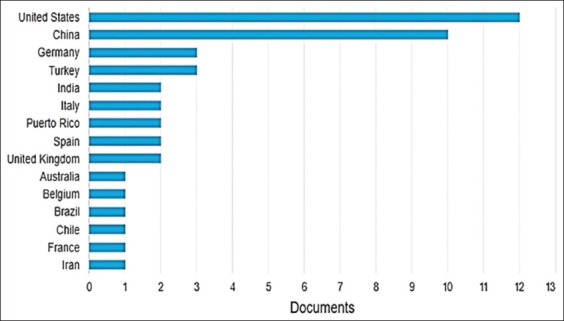
Country-wise distribution of publications on pain treatment in endometriosis in experimental rat models, 2012–2022, Bibliometric Review, 2023.

A total of 15 countries were identified in the author affiliation section of the publications included in this review. The United States had the highest number of affiliations (12), followed by China (10). Germany and Turkey had three publications each (Figure-2).

**Figure-3:**
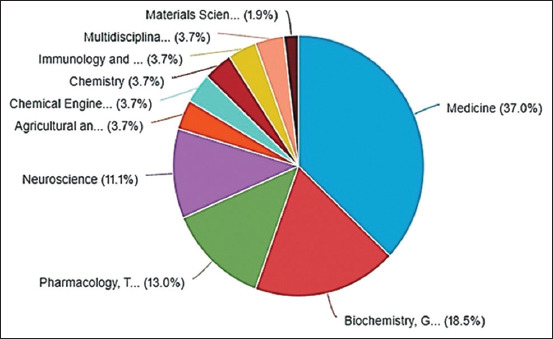
Subject area distribution of publications on pain treatment in endometriosis in experimental rat models, 2012–2022, Bibliometric Review, 2023.

In terms of the areas with the highest publication output, 10 were identified, with medicine (37.0%, 20 publications) ranking first, followed by biochemistry, genetics, and molecular biology (18.5%, 10 publications); pharmacy, pharmacology, and toxicology (13.0%, 7 publications); and then neuroscience (11.1%, 6 publications) (Figure-3).

### Citations and index analysis

Table-2 presents the number of citations per year for each publication and the cumulative citations for the top 5 publications from 2012 to 2022, totaling 402 citations. The most cited journals are also highlighted along with their respective indicators: American Journal of Pathology (80 citations, CiteScore 8.6/SJR 1.619 SNIP 1.227), Journal of Visualized Experiments (44 citations, CiteScore 2.5/SJR 0.504/SNIP 0.449), Frontiers in Pharmacology (38 citations, CiteScore 6.6/SJR 1.143/SNIP 1.388), Reproductive Sciences (25 citations, CiteScore 4.2/SJR 0.642/SNIP 0.770), and Pain (25 citations, CiteScore 10.6/SJR 2.135/SNIP 2.172).

**Table-2:** Citations of the top 5 publications on pain management in endometriosis in experimental rat models, 2012–2022.

Article title	Journal	Number of citations						

		<2018	2018	2019	2020	2021	2022	Total
						
		71	43	50	67	75	96	402
Estradiol is a Critical Mediator of Macrophage- Nerve Cross Talk in Peritoneal Endometriosis	American Journal of Pathology	14	12	13	15	11	16	80
Mouse model of surgically-induced endometriosis by auto-transplantation of uterine tissue	J. of Visualized Experiments	10	5	12	5	5	7	44
Co-micronized palmitoylethanolamide/polydatin treatment causes endometriotic lesion regression in a rodent model of induced endometriosis	Frontiers in Pharmacology	4	6	6	5	11	6	38
Bentamapimod (JNK inhibitor AS602801) induces regression of endometriotic lesions in animal models	Reproductive Sciences	2	4	3	6	11	0	25
Oxidation-sensitive nociception involved in endometriosis-associated pain	Pain	8	2	4	3	3	5	25

**Figure-4:**
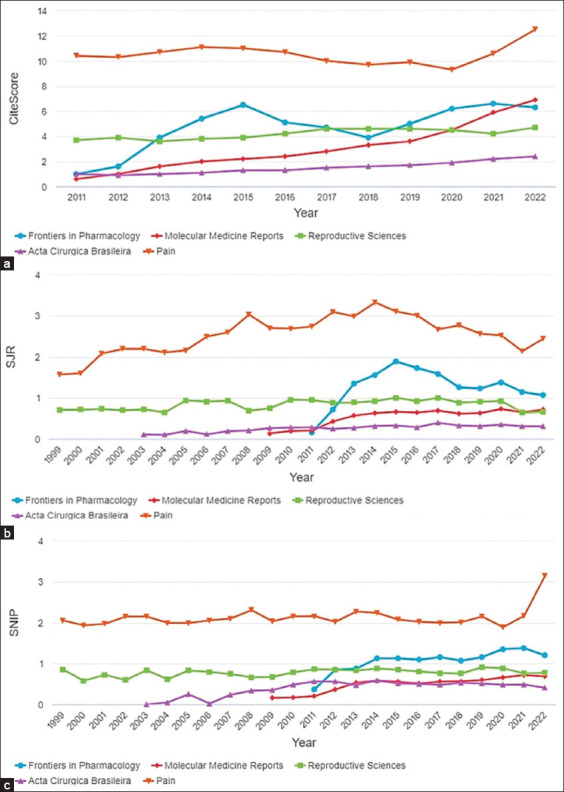
Indicators of publications on pain treatment in endometriosis in experimental rat models, 2012–2022, Bibliometric Review, 2023. (a) CiteScore, (b) SCImago Journal Rank, and (c) Source-normalized impact per paper.

Figure-4 presents the CiteScore (A), SJR (B), and SNIP (C) graphs, showing the comparison of the citation and impact factor related to endometriosis, rats, treatment, and pain. As shown in figure, the journals “Pain” and “Frontiers in Pharmacology” consistently obtained the highest scores and metrics over the years.

The use of CiteScore is a straightforward way to measure the impact of citation sources, such as journals. It is based on the number of citations received by documents published in a journal over a 4-year period divided by the number of indexed documents of the same type in the Scopus database published in those same 4 years. The SJR graph represents the average weighted citations received in the selected year by documents published in the selected journal in the previous 3 years. The SNIP graph, on the other hand, provides a corrective metric to account for differences in citation potential across different fields.

**Figure-5:**
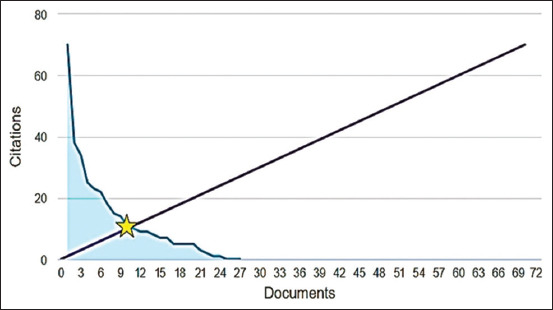
H-index of publications on pain treatment in endometriosis in experimental rat models, 2012–2022, Bibliometric Review, 2023.

**Figure-6:**
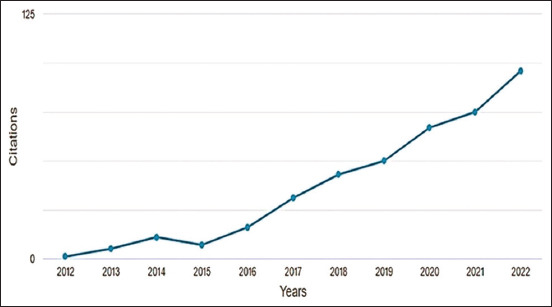
Citations of publications on pain treatment in endometriosis in experimental rat models, 2012–2022, Bibliometric Review, 2023.

Figure-5 presents a 45° line, which represents the relationship between publishing and being cited. Figure-5 emphasizes that of the 30 documents considered for the H-index, 13 have been cited at least 13 times. An overview of the selected documents showedaincreasing trend of citations for publications from 2016 (16 citations), 2017 (31 citations), 2018 (43 citations), 2019 (50 citations), 2020 (67 citations), 2021 (75 citations), and 2022 (96 citations) (Figure-6).

### Co-occurrence and keyword density analysis

**Figure-7:**
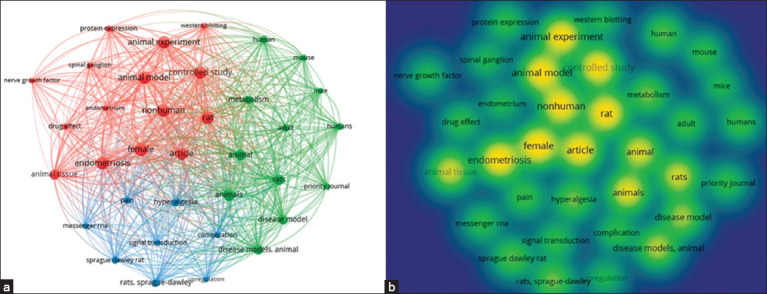
(a) Co-occurrence network of keywords and (b) word density visualization in publications on pain treatment in endometriosis in experimental rat models, 2012–2022, Bibliometric Review, 2023.

To elucidate the relationship between the most cited references, co-occurring keywords, clusters, and bursts, with a focus on endometriosis pain management using rat experimental models, the network of co-occurring keywords (A) and the density of words or “hotspots” (B) were analyzed. A total of 542 terms were mapped, and the selection criterion required a minimum occurrence of 5 times in the publications, resulting in 35 words. Among these keywords, eight stood out: “animal experiment,” “animal model,” “article,” “controlled study,” “endometriosis,” “female,” “nonhuman,” and “rat” (Figure-7).

Visualization of the co-occurrence analysis of keywords presents colored circles indicating the frequency of occurrence of these words. The lines connecting the nodes represent their co-occurrence in the same publication, with shorter distances denoting a higher occurrence of the two keywords. The hotspots are represented by the intensity and size of the colored circles, with intense yellow denoting significance.

### Group analysis of authors and the most cited authors

**Figure-8:**
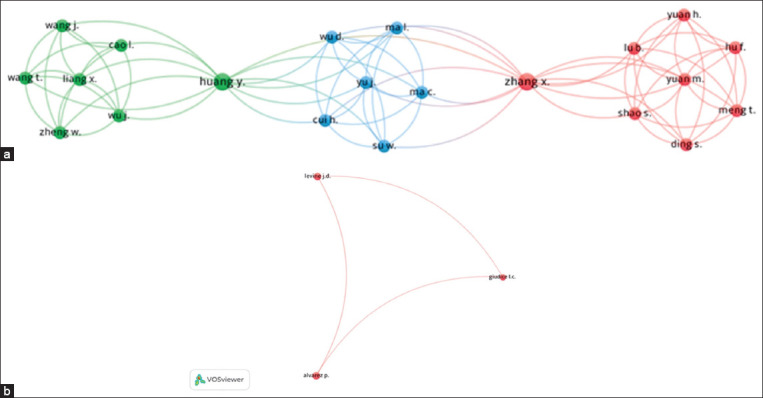
(a) Visualization of groups of authors, and (b) visualization of the most cited authors in publications on pain treatment in endometriosis in experimental rat models, 2012–2022, Bibliometric Review, 2023.

In the analysis of authors and coauthors, 135 names and three potential author groups were identified. By examining the strength of connections among these 135 names, it was found that the largest connected cluster consisted of eight names, which are highlighted in red (A). Furthermore, regarding the most cited and frequently copublished authors, three names were present in the studies: Alvarez and Levine [16], Alvarez *et al*. [17], and Alvarez *et al*. [18], indicating academic collaboration (Figure-8).

## Discussion

This bibliometric review study provides a comprehensive overview of the literature on endometriosis pain management in experimental rat models over the past decade.The most recent and globally relevant academic contributions in this field were identified by analyzing the available publicationsd. The analysis revealed a limited number of publications in indexed databases, with a total of 30 articles identified. This translates to an average of only three articles per year during the 10-year period, with a peak of five publications observed in 2021. This scarcity of publications may be attributed to the complex etiopathogenesis of endometriosis and challenges associated with its treatment. It could also indicate a need for increased investment and financial collaboration from advanced research centers to conduct further research in this area [4, 5, 13]. Evidently, there is a pressing need for more extensive studies focusing on endometriosis pain management.

Notable disparities also exist in the geographical distribution of published studies, with the United States and China emerging as the leading contributors in terms of research output, technical and scientific advancements, and financial collaborations [43]. On the other hand, Latin America, Africa, and Oceania demonstrate a significant gap in this field. This discrepancy may be attributed to the unfavorable socioeconomic profiles of these developing countries along with limited access to healthcare services. Furthermore, the average diagnostic delay of 7 years for endometriosis in these regions and inadequate investment in medical education [44, 45] could further contribute to the scarcity of research publications.

It is important to consider the significant differences in technical cooperation and funding from institutes and universities between countries such as the United States and Brazil. Developed countries often benefit from extensive collaborations and financial support in various research areas. This promotes technical and scientific advancements and generates a greater interest in publication in high-impact journals. Contrarily, developing countries are frequently underrepresented in leading medical journals [45, 46].

As for the most prominent articles identified in this review, it is noteworthy that five received the highest number of citations, ranging from 80 to 25. While categorized within the field of medicine, these publications did not achieve significant international and multidisciplinary visibility. This finding is consistent with other bibliometric reviews that have also highlighted the challenges associated with pain-related research [11, 46, 47].

Among the analyzed articles, one intriguing aspect is the strong association between the terms “endometriosis” and “pain.” Pain represents the most prominent and defining manifestation of the disease, often causing significant discomfort and disability. Paradoxically, studies focused on advancing our understanding of endometriosis pain management are lacking. At present, the efficacy of treatments is mainly assessed based on pain improvement or control and fertility rates, to enhance the overall quality of life of the affected women [48, 49].

Another notable finding of this bibliometricreview is the limited impact of the publications, as evidenced by the H-index. However, when considering other citation metrics and impact scores, such as CiteScore, SJR, and SNIP, it becomes apparent that they mainly target specific aspects of endometriosis and pain research. Interestingly, the most significant publications were predominantly found in journals dedicated to the medical field of gynecology and obstetrics. Only one of the five key articles was published in a specialized pain journal. There are several factors and barriers that contribute to this low impact of publications.

First, many journals tend to prioritize specific, innovative, and contemporary topics; thus, studies on endometriosis pain management that do not align with the latest trends may be overlooked. Second, the quality of publications plays a crucial role in determining their reach and acceptance in specialized and high-impact journals. Publications that fail to meet the required standards may struggle to gain visibility in prominent outlets. Furthermore, sufficient incentives for research in this field appear to be lacking, hindering the overall progress and recognition of studies.

Another significant aspect influencing publication impact is the need for greater financial and technological investment. Unfortunately, limited financial support and access to advanced technologies often hinder the execution of rigorous research, thereby affecting the quality and impact of publications. Notably, the impact factor of a journal does not necessarily reflect the intrinsic quality of an individual article [11–46]. In addition, research resources are often directed toward developing high-cost technologies and treatments. This makes the implementation of study results in low-income countries challenging, as they lack the necessary resources to adopt the advancements [50].

During word density analysis, a notable absence of the term “treatment” was observed, both in the abstracts and titles of the publications. However, it is noteworthy that the term “treatment” played a crucial role in the guidance and shaping of this review. Choosing appropriate terms that accurately reflect the research objectives is of utmost importance as they provide a clear direction for readers and ensure the efficacy of search strategies used in databases [50].

In the two author network mappings conducted, distinct patterns were observed. First, a larger network consisting of Chinese researchers mainly affiliated with the same institution or specific region was identified. This network demonstrated branching into smaller groups; however, it was characterized by the presence of key researchers in each group, thereby consolidating the number of research groups. Contrarily, the other network comprised North American authors, which had a smaller number of authors but a higher number of citations in the field of pain. This observation could potentially be attributed to regional isolation among researchers, limited opportunities for knowledge exchange, and a lack of interinstitutional support [50].

Notably, the maps did not include references to influential researchers from countries such as Germany and the United Kingdom. This absence can be attributed to the low production of research on the subject in such countries, as it is not uncommon for bibliometric reviews to observe such trends [14, 50].

This study demonstrates the quantity and quality of publications regarding endometriosis pain management in experimental models in the past 10 years, where the method used for research is innovative, as we have not observed any bibliometric analysis developed for field in the scientific literature. Therefore, this study is important as it quantifies the latest publications on this topic, presenting which institutions and countries are developing research on the topic and which countries are financing these researches.

For the medical field and clinicians, the study of endometriosis pain management in experimental models helps elucidate its pathogenesis, which is multicausal, and favor diagnosis, which is late in most cases, in addition to seeking more effective treatment to control pain. This study makes it possible to translate the description of endometriosis pain, as it provides scientific evidence for translating preclinical studies to clinical ones.

## Conclusion

This study has certain limitations, including the limited number of references available for data comparison, the focus solely on studies conducted in rats, and the restriction to publications in the English language. Consequently, it is evident that studies on endometriosis pain management, specifically in experimental rat models, are scare. Thus, it is imperative to promote further research across various fields of knowledge to address this issue, encouraging funding and international collaboration. Only through these means can we establish a robust foundation of information and generate more substantial findings regarding effective treatments for controlling this type of pain. It is crucial to address this research gap, as endometriosis significantly impacts the quality of life of the affected women.

## Authors’ Contributions

RAL, IBJS, GMF, TCMMP, and ICA: Designed the study, performed data analysis, and manuscript preparation. EMS, MSSC, RCC, ASPF, and JBSG: Coordinated the study, wrote, and revised the manuscript. All authors have read, reviewed, and approved the final manuscript.
